# BMI is a poor predictor of adiposity in young overweight and obese children

**DOI:** 10.1186/s12887-017-0891-z

**Published:** 2017-06-02

**Authors:** Cassandra Vanderwall, R. Randall Clark, Jens Eickhoff, Aaron L. Carrel

**Affiliations:** 1 University of Wisconsin, Madison, WI USA; 2 UW Health, University Hospital, 600 Highland Ave, Madison, WI 53792 USA

**Keywords:** Body mass index, Childhood obesity, Dual X-Ray absorptiometry, Body composition

## Abstract

**Background:**

The body mass index (BMI) is a simple and widely utilized screening tool for obesity in children and adults. The purpose of this investigation was to evaluate if BMI could predict total fat mass (TFM) and percent body fat (%FAT) in a sample of overweight and obese children.

**Methods:**

In this observational study, body composition was measured by dual energy x-ray absorptiometry (DXA) in 663 male and female overweight and obese children at baseline within a multidisciplinary, pediatric fitness clinic at an academic medical center. Univariate and multivariate regression analyses were conducted to evaluate whether BMI z-score (BMIz) predicts TFM or %FAT.

**Results:**

The BMIz, sex and age of subjects were identified as significant predictors for both TFM and %FAT. In subjects younger than 9 years, the BMIz was a weak to moderate predictor for both TFM (R^2^ = 0.03 for males and 0.26 for females) and %FAT (R^2^ = 0.22 for males and 0.38 for females). For subjects between 9 and 18 years, the BMIz was a strong predictor for TFM (R^2^ between 0.57 and 0.73) while BMIz remained only moderately predictive for %FAT (R^2^ between 0.22 and 0.42).

**Conclusions:**

These findings advance the understanding of the utility and limitations of BMI in children and adolescents. In youth (9-18y), BMIz is a strong predictor for TFM, but a weaker predictor of relative body fat (%FAT). In children younger than 9y, BMIz is only a weak to moderate predictor for both TFM and %FAT. This study cautions the use of BMIz as a predictor of %FAT in children younger than 9 years.

## Background

Childhood obesity is a global public health crisis [1, 2] and obesity in the United States has more than doubled in children and quadrupled in adolescents over the last 30 years [3, 4]. At present, more than one-third of children and adolescents in the United States are overweight or obese, more than 17% of these youth are obese [3]. Childhood obesity is associated with cardiovascular disease, hypertension, insulin resistance and type 2 diabetes, asthma, obstructive sleep apnea, psychosocial problems, decreased quality of life, and increased likelihood of becoming obese adults [3, 5–15]. Morbidity and mortality risk may vary between different racial and Hispanic origin groups at the same body mass index (BMI) [16, 17]. Adiposity is an independent risk factor for insulin resistance and a strong predictor of morbidity [18–21]. Therefore, directly assessing body fat is a key strategy for preventative and therapeutic intervention of childhood obesity [18, 22].

Obesity, or having excess body fat [23], can be defined using cut points of BMI; the ratio of an individual’s weight to height squared (kg/m^2^). The BMI varies with age in children and thus BMI values are compared with age- and sex-specific references. For children and adolescents aged 2 to 19 years, BMI is plotted on the sex-specific, Centers for Disease Control and Prevention (CDC) growth chart to identify the BMI-for-age percentile. Childhood obesity is defined as a BMI at or above the 95th percentile on the BMI-for-Age growth chart The BMI-for-age percentile is calculated based on a reference population [22, 24]. The indirect relationship between BMI and measures of adiposity has been established but varies according to sex, age, and race-ethnicity [16, 17].

The literature also varies in the strength of the association between BMI and body composition variables [24–26]. Therefore, the purpose of this investigation was to evaluate the relationships between BMIz, total fat mass (TFM) and percent body fat (%FAT) using dual energy x-ray absorptiometry (DXA) in a sample of overweight and obese children. This study evaluated the relationship between BMIz and TFM, as well as, BMIz and %FAT as determined by DXA in four age categories of overweight and obese children: 4–9, 9–11, 12–14, and 15–18 years.

Traditional anthropometric measures (weight, waist circumference, BMI) used to evaluate and track changes in body composition can misclassify patients and may not accurately assess significant changes in body composition over time. The most common clinical body composition tools include waist circumference, skinfold calipers, bio-electrical impedance analysis (BIA), air displacement plethysmography (ADP), hydrodensitometry, and DXA [27, 28]. Due to ease of acquisition, the most widely used clinical outcome variable is BMI. Historically, BMI has been accepted as the standard clinical screening tool for youth to determine their risk status for disease states related to weight and adiposity [22, 23]. However, the relationships between BMI and laboratory measurement of body fat and lean tissue mass are not clear in today’s generation of overweight and obese youth. Primary care providers play a pivotal role in the process of preventing, identifying and treating childhood obesity and associated co-morbidities [29–34] and frequently use BMI to screen for excess body fat relative to body weight. It is unclear whether BMI can be utilized to monitor changes resulting from weight management interventions designed to improve body composition in this population. Therefore, this study evaluated the effectiveness of BMI to predict TFM and %FAT by DXA in overweight and obese youth.

## Methods

All subjects were overweight or obese boys and girls (ages 4–18 years) evaluated as part of their routine clinical care at a multidisciplinary weight management program within an academic medical center. Anthropometric and body composition measurements were collected at the same initial encounter. Measurement procedures were performed and analyzed by the same investigators. Height was measured with a wall-mounted stadiometer to the nearest 0.1 cm. Weight was measured on a calibrated beam balance platform scale to the nearest 0.1 kg. BMI z-score (BMIz) and BMI-for-age percentiles were computed using the CDC reference values.

The body composition values of total body bone, muscle and fat mass, as well as, %FAT were measured by DXA. Whole body scans were performed using the Norland XR-36 whole body bone densitometer (Norland Corporation, Ft. Atkinson, Wisconsin USA) and tissue masses were analyzed using software version 3.7.4/2.1.0. All subjects were positioned in the supine position and scanned by the same investigator. Subjects removed metal objects or clothing containing metal components and wore only workout shorts and t-shirt for the scan procedure. Each scan session was preceded by a calibration routine using multiple quality control phantoms that simulate soft tissue and bone. Based on 18 scans of 6 subjects using the XR-36 whole body procedures the total body coefficients of variation (CV) are as follows: soft tissue mass 0.2%, total body mass 0.2%, lean body mass 1.0%, fat mass 2.5%, percent fat 2.4% and total BMC 0.9%. The Norland XR-36 has been previously validated for measurement of body composition against multi-component models [35–37]. Study procedures were approved by the Health Sciences Human Subjects Committee at the University of Wisconsin- Madison.

All baseline characteristics were summarized in terms of means (SD) or frequencies and percentages. Univariate and multivariate regression analyses were conducted to evaluate the association between BMIz and markers of body composition, including TFM and %FAT. The univariate analyses were stratified by gender and designated age groups: 4–9 years, 9–11 years, 12–14 years, and 15–18 years Multiple regression analysis models were created with TFM and %FAT as dependent variables and BMI z-score and age as independent variables. Slope parameter estimates were reported along with the corresponding 95% confidence intervals (CIs). Furthermore, moving average regression analyses of TFM on BMIz and relative %FAT on BMIz across the continuous age range (4–18 years) with age windows of +/−1 year were conducted in order to visually display how the association between TFM, relative fat and BMIz changes with age. The corresponding Rw^2^ values were calculated and plotted using the smoothing spline method. Statistical analyses were conducted using SAS software version 9.4 (SAS Institute Inc., Cary NC). All reported *P*-values are two-sided and *P* < 0.05 was used to define statistical significance.

## Results

Subjects were 663 overweight and obese boys and girls (49% male) with a mean (SD) age of 11.7 (3.3) years (range 4–18 years), BMI of 30.2 kg/m^2^ (6.5) and BMIz of 2.2 (0.5). Mean body composition values for all subjects were a TFM of 36.1 (14.2) kg and %FAT of 39.3% (5.2) in the sample (Table 1). The majority (90%) of the subjects were obese of which 279 (47%) were severely obese with a BMI-for-age above the 99th percentile (Table 1). The TFM and %FAT were significantly higher in severely obese subjects (BMI-for-age > 99th percentile) when compared to subjects within the 85th to 99th BMI percentile range (*p* < 0.001) (Table 2).

**Table 1:** Subject characteristics

	Male		Female		Overall	
	(*N* = 325)		(*N* = 338)		(*N* = 663)	
	*N*	%	*N*	%	*N*	%
Age (years)						
4–9	66	20%	50	15%	116	18%
9–11	101	31%	106	31%	207	31%
12–14	90	28%	104	31%	194	29%
15–18	68	21%	78	23%	146	22%
BMI-for-Age percentile						
85 to 95th	25	8%	38	11%	63	10%
95 to 99th	147	45%	174	51%	321	48%
> 99th	153	47%	126	37%	279	42%
BMI (kg/m^2^)						
Mean ± SD	29.8 ± 6.1		30.7 ± 6.9		30.3 ± 6.5	
BMI z-score						
Mean ± SD	2.3 ± 0.5		2.2 ± 0.4		2.2 ± 0.5	
Total Fat Mass, TFM (kg)						
Mean ± SD	34.4 ± 13.1		37.7 ± 15.0		36.1 ± 14.2	
Percent Body Fat, %FAT (%)						
Mean ± SD	38.3 ± 5.6		38.3 ± 5.6		39.3 ± 5.2

**Table 2:** Mean ± SD total fat mass (TFM) and percent body fat (%FAT) by BMI-for-age percentiles and sex

	BMI percentile				
Sex	Body Fat Measure	85th–95th	95th–99th	>99th	*p*-value
Male	TFM (kg)	23.0 ± 5.7	30.6 ± 8.2	39.8 ± 15.2	<0.001
	%FAT	33.9 ± 5.9	36.6 ± 5.4	40.7 ± 4.6	<0.001
Female	TFM (kg)	26.5 ± 7.1	34.7 ± 10.7	45.1 ± 18.1	<0.001
	%FAT	35.7 ± 4.2	39.0 ± 3.8	43.1 ± 4.2	<0.001

**Fig. 1:**
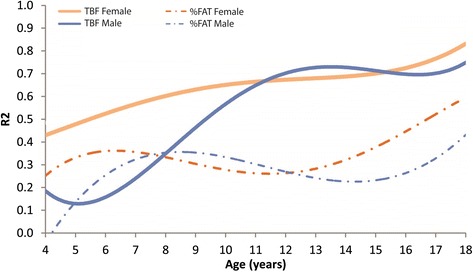
Regression analysis (R^2^) for moving average across continuous age range of total fat mass (TFM) on the BMI z-score (BMIz) and relative fat (%FAT) on BMIz, stratified by sex

In the multivariate regression analysis, BMIz (*p* < 0.001), sex (*p* < 0.001) and age (*p* = 0.01) were identified as independent predictors for TFM. Furthermore, a significant interaction effect between age and BMIz was detected (*p* < 0.001). For %FAT, only BMIz (*p* < 0.001) and sex (*p* < 0.001) were identified as significant predictors. The results of the age-stratified analysis are shown in Table 3 and visually displayed in Fig. 1 for males and females. In subjects younger than 9 years, BMIz was identified as a weak to moderately strong predictor for both TFM (R^2^ = 0.03 for males and 0.26 for females) and %FAT (R^2^ = 0.22 for males and 0.38 for females). For subjects between 9 and 18 years, on the other hand, BMIz was identified as a strong predictor for TFM (R^2^ between 0.57 and 0.73) while BMIz remained only weakly to moderately predictive for %FAT (R^2^ between 0.22 and 0.42) for both males and females (Table 3). The partial correlation coefficient between BMIz and TFM was 0.67 (95% CI: 0.60–0.72) for males and 0.82 (95% CI: 0.78–0.85) for females after adjusting for sex and age while the partial correlation coefficient between BMIz and %FAT was 0.39 (95% CI: 0.30–0.48) for males and 0.60 (95% CI: 0.52–0.66) for females. These results indicate a relationship between BMIz and TFM, as well as, BMIz and %FAT varying by age and sex.

**Table 3:** Univariate and multivariate regression analysis for predicting total fat mass (TFM) and percent body fat (%FAT) on BMI z-score in an overweight and obese pediatric population (4–18 years), stratified by sex and age groups

		Outcome: Total Fat Mass, TFM (kg)				Outcome: Percent Body Fat, %FAT (%)			
Age (years)	Gender	β (Slope)	95% CI for β	*P*-value	R^2^	β (Slope)	95% CI for β	*P*-value	R^2^
4-9^a^	Male	1.9	−0.8–4.6	0.161	0.03^c^	3.7	1.9–5.4	<0.001	0.22^d^
	Female	6.9	3.6–10.3	<0.001	0.26^d^	7.0	5.2–8.8	<0.001	0.38^d^
9–11^a^	Male	18.1	15.3–20.9	<0.001	0.63^e^	7.0	5.2–8.8	<0.001	0.38^d^
	Female	17.1	14.2–20.0	<0.001	0.57^e^	6.7	4.4–9.1	<0.001	0.42^d^
12–14^a^	Male	27.0	23.2–30.9	<0.001	0.69^e^	7.3	4.4–10.2	<0.001	0.22^d^
	Female	25.7	22.2–29.2	<0.001	0.67^e^	6.1	4.4–8.0	<0.001	0.29^d^
15–18^a^	Male	26.4	22.5–30.3	<0.001	0.73^e^	7.2	4.2–10.1	<0.001	0.26^d^
	Female	30.4	26.2–34.6	<0.001	0.73^e^	6.0	4.0–7.9	<0.001	0.26^d^
Overall^b^	Male	15.1	13.3–17.0	<0.001	0.66^e^	4.4	3.3–5.5	<0.001	0.29^d^
	Female	21.9	20.3–23.6	<0.001	0.82^e^	6.8	5.8–7.8	<0.001	0.37^d^

^a^Univariate regression analysis of TBF and %FAT on BMIz

^b^Multivariate regression analysis of TBF and %FAT on BMIz and age

^c^BMIz was non-predictive of this outcome variable

^d^BMIz was a moderate predictor of this outcome variable

^e^BMIz was a strong predictor of this outcome variable

## Discussion

The BMI is widely used as a screening tool as a proxy for weight-related health risk because high BMI values may reflect excess adiposity. However, BMI does not estimate body composition and cannot differentiate between fat and muscle in children. Our study demonstrates that age has a strong interaction with %FAT, but in children younger than 9 years, the BMIz is a weak predictor for both TFM and %FAT. The BMIz is only a weak predictor for TFM and %FAT in young children, less than 9 years of age. These data, however, are different for older children. The BMIz is a strong predictor of TFM in children and adolescents over the age of 9 years. These results have strong implications for the use and reliance on the BMI for screening and monitoring weight-related changes in overweight and obese youth.

It is important to consider the difference between TFM and %FAT. Total fat mass is the absolute fat mass for that individual. The TFM value does not identify an individual’s relative fat, or the amount of fat in relation to their bone, muscle and total body mass. While it has been shown that DXA is a more accurate measure for adiposity, [38, 39] it may not be practical on a large scale due to cost and resource constraints, and is not currently available and used in the greater community [40]. However, many clinicians continue to utilize BMI as a screening tool for obesity and weight-related disease states based on the assumption that a high BMI equals a high degree of adiposity. However, the results of the current study using DXA, indicate that BMI is not diagnostic of the degree of body fatness in younger children. Because childhood obesity has been identified as a global public health crisis [1, 2], clinicians should be aware of weaknesses in utilizing BMI to estimate excess body fat in younger children.

Flegal [16] utilized NHANES (1999–2004) data to assess the performance of the standard BMI-for-age percentile categories relative to the prevalence of excess adiposity (%FAT) using DXA in 8,821children ages 8 to 19 years of age. They concluded that a narrow range of the BMI-for-age percentiles identify individuals with both a high BMI and excess adiposity and large differences in the prevalence in children and adolescents with intermediate BMI-for-age percentile ranges and high adiposity. Flegal, et al. encourages caution when interpreting comparisons of high BMI ranges in terms of adiposity, by race-ethnicity, as well as, in the interpretation of the relationship between BMI and adiposity in children with intermediate BMI ranges. The present study only examined overweight and obese children and adolescents and the present results support Flegal’s findings that BMI maintains a weak relationship with relative body fat (%FAT) in overweight and obese children and adolescents and also cautions the use of BMI as a predictor of %FAT in children younger than 9 years.

Pietrobelli [41] found that BMI was strongly associated with TFM (R^2^ = 0.85 and 0.89 for boys and girls, respectively) and %FAT (R^2^ = 0.63 and 0.69 for boys and girls, respectively). While Pietrobelli concluded that the association between BMI and adiposity is consistent across the age spectrum, our data does not support this in children less than 9 years of age. Their sample was comprised of healthy children with a mean BMI of 23.8 kg/m^2^ which was lower than the mean BMI for the present sample (30.2 kg/m^2^). The Pietrobelli work represents earlier exploratory efforts to understand and associate BMI with more robust measures of body fat. The new CDC BMI growth charts utilize percentiles due to the fact that simple BMI does not represent relative adiposity very well; BMI z-scores must be calculated and used when working with children and adolescents [42].

Our conclusions align with Katzmarzyk [24]; we recognize that healthcare practitioners should also exercise caution when comparing BMI across race-ethnicity groups. Additionally, BMI may misclassify some segments of the pediatric population. Clinicians should be careful when utilizing BMI alone to classify an individual’s %FAT [26, 28, 40, 43].

The present assessment is novel because it 1) uses an analysis stratified by age to evaluate the limitation of BMI and BMIz for estimating adiposity (TFM and %FAT) in overweight and obese children, 2) identifies the non-predictive nature of BMIz relative to TFM in younger children (4–9 years) and 3) utilizes DXA for body fat to evaluate these relationships. A strength of the current study was the age-stratified analysis in a large cohort (*n* = 663) of overweight and obese children. A limitation of the study and area of future investigation would be to identify the difference in correlations or associations by race-ethnicity. Another potential area of future research is to investigate if the BMIz is a valid tool for monitoring significant changes in a pediatric subject’s TFM, lean mass and %FAT over time when compared to DXA.

## Conclusions

These findings advance the understanding of the utility and limitations of BMI in children. This study utilized multivariate modeling to assess the relationship between BMIz with TFM and %FAT using DXA in an overweight and obese pediatric population (4–18 years) stratified by age. These data indicate that there is a strong interaction effect for the association between BMIz and TFM with respect to age. In overweight and obese youth, aged 9 to 18 years, BMI z-score is a strong predictor for TFM, but only a weak-to-moderate predictor of %FAT. In overweight and obese children younger than 9 years, the BMIz is a weak predictor for both TFM and %FAT. Under the conditions of the study, these data indicate a relationship between BMI and TFM, a weaker association with relative body fat (%FAT), and demonstrate the limitation of using BMIz as a predictor of %FAT in overweight and obese children under 9 years of age.

## Abbreviations

%FAT: Total percent body fat
BMI: Body mass index
BMIz: Body mass index z-score
DXA: Dual energy x-ray absorptiometry
TFM: Total fat mass
